# Pathologic fracture and hardware failure in *Streptococcus anginosus* femoral osteomyelitis: Case report

**DOI:** 10.1016/j.amsu.2021.102478

**Published:** 2021-06-09

**Authors:** John E. Stillson, Connor M. Bunch, Anthony V. Thomas, Nicolas Mjaess, Joseph A. Dynako, Andres S. Piscoya, Joel M. Post, Brian L. Ratigan, Zachary H. Goldstein, Mark M. Walsh

**Affiliations:** a Indiana University School of Medicine South Bend Campus, Notre Dame, IN, USA; b Department of Emergency Medicine, Saint Joseph Regional Medical Center, Mishawaka, IN, USA; c Department of Orthopedic Surgery & Rehabilitation, University of Mississippi Medical Center, Jackson, MS, USA; d Department of Orthopaedic Surgery, Walter Reed National Military Medical Center, Bethesda, MD, USA; e Beacon Bone & Joint Specialists Navarre, Memorial Hospital, South Bend, IN, USA; f South Bend Orthopaedics, South Bend, IN, USA; g Department of Orthopedic Surgery, Indiana University Health University Hospital, Indianapolis, IN, USA

**Keywords:** Orthopedics, Spontaneous fractures, Osteomyelitis, Femur, Streptococcus anginosus, Case report, CAD, coronary artery disease, CYP450, cytochrome-P450, DM, diabetes mellitus, HLD, hyperlipidemia, HTN, hypertension, IV, intravenous, MRI, magnetic resonance image, RIA, reamer-irrigator-aspirator

## Abstract

**Introduction:**

Pathologic fracture of the femur due to *Streptococcus anginosus* osteomyelitis has rarely been described. With limited evidence for treating *S. anginosus* osteomyelitis, the orthopaedic surgeon is presented with a difficult treatment decision at index presentation. Presented here is a case of failed conservative management, diagnostic dilemma, failed hardware stabilization, and definitive surgical treatment resulting in good clinical outcome.

**Case presentation:**

A 69-year-old male experienced acute right thigh pain, edema, and erythema after dental treatment 17 days prior. He was diagnosed with right femoral diaphyseal osteomyelitis and Brodie's abscess. Blood cultures grew *S. anginosus*, but all site-specific tissue cultures resulted negative. Initial management consisted of intravenous antibiotic therapy and percutaneous abscess drainage. Months later, the patient sustained a displaced pathologic fracture of the diaphyseal femur and there was concern for neoplasm, but biopsies were negative. Stabilization was attempted with a lateral plate and screws. This hardware catastrophically failed in the setting of an oligotrophic femoral nonunion. Ultimately, the patient was successfully treated with an intramedullary nail coated with antibiotic-impregnated cement. Twelve months later, the patient achieved clinical and radiographic healing with no evidence of relapse of his osteomyelitis.

**Clinical discussion:**

Conservative management of *S. anginosus* femoral osteomyelitis was inadequate and corroborates the existing literature. *S. anginosus* osteomyelitis and pyomyositis may be most optimally treated aggressively with early surgical intervention.

**Conclusion:**

Early surgical debridement and stabilization of the compromised bone with an antibiotic coated intramedullary nail following medullary reaming may prevent pathologic fracture, eradicate infection, and achieve predictable outcomes.

## Highlights

• *S. anginosus* femoral osteomyelitis is rare and difficult to diagnose and treat.
• Conservative measures failed, and pathologic fracture later ensued.
• Although sparse, existing reports suggest early surgical management is indicated.
• This report provides more evidence for early and aggressive surgical management.
• Good outcome was achieved with antibiotic coated intramedullary nailing.

## 1 Introduction

Pathologic fracture of the femur due to *Streptococcus anginosus* osteomyelitis has been described rarely with no consensus on definitive therapy (Table 1) [[1], [2], [3], [4]]. Reports are scarce because hematogenous femoral osteomyelitis rarely inflicts the immunocompetent adult, and *S. anginosus* is a particularly rare pathogen [5]. Here, this report describes a case of failed initial conservative management that was later complicated by pathologic fracture and catastrophic implant failure after diagnostic workup of the fracture resulted in negative tissue biopsies for neoplasia or an infectious agent. Significantly, definitive surgical management and good outcome was achieved despite early therapeutic failures. Diagnostic and therapeutic dilemmas of *S. anginosus* osteomyelitis are discussed in the context of the sparse existing literature, and this report corroborates the existing evidence for early, aggressive surgical intervention. This case report has been reported in line with the SCARE Criteria [6].

**Table 1:** Review of current literature on *S. anginosus* induced pathologic fracture of the femur.

Report	Demographic	Risk Factors for Bone Infection and Fracture	Surgical Intervention	Outcome
Thein et al. [1]	56-y/o male	- CAD - DM - HTN	- Fracture care: Antibiotic-coated intramedullary nail and external fixator - Follow-up care: Intramedullary femoral nail exchange	No signs of infection, but suffered pain and difficulty ambulating at 17 months follow-up
Vajapey et al. [2]	52-y/o male	- Alcoholism - Dental abscesses discovered during hospitalization	- Fracture care: Reaming of femoral intramedullary canal with RIA, and antibiotic-coated nail - Follow-up care: Exchange of antibiotic-coated nails *x*2 prior to definitive nail placement	Definitive intramedullary nail retained at 12 months follow-up with full weight-bearing
Krebs et al. [3]	53-y/o male	- DM - CYP450 induction - Bacteremia	- Fracture care: Reamed cephalomedullary nail with a distal locking screw - Follow-up care: RIA *x*2, antibiotic-impregnated cement rod and external fixator *x*2	Removal of antibiotic- impregnated cement rod and external fixator after six weeks from previous surgery with no residual infection
Janssen et al. [4]	57-y/o male	- Alcoholism - HTN	- Fracture care: external fixator - Follow-up care: n/a	Severe septic shock and expiration
This report	69-y/o male	- Former alcoholic - Former tobacco use - Dental surgery 17 days prior to symptom onset - HTN - HLD	- Fracture care: Stabilization with internal plate - Follow-up care: RIA, one-stage procedure comprising antibiotic-coated intramedullary nail, and bone autograft	Definitive intramedullary nail retained at 12 months with full weight-bearing, no pain, and no signs of infection

## 2 Case Presentation

A 69-year-old Caucasian male who was a former alcoholic and former smoker presented to the emergency department with a chief concern of right lower back pain radiating down the right thigh to the level of the knee for three weeks. There was no history of trauma or recent illness. However, the patient had received a routine dental cleaning 17 days prior to the onset of symptoms. Vital signs at index were remarkable for tachycardia and hypertension. Physical exam revealed diffuse swelling of the right thigh, pitting edema of the extremity, and some erythema and warmth over the medial thigh. No crepitus was noted. Range of motion was normal at the right hip and knee.

Plain radiographs of the right femur were unremarkable. Upon hospital admission, laboratory studies revealed leukocytosis, elevated alkaline phosphatase, elevated liver transaminases, low albumin, elevated *C*-reactive protein, and elevated erythrocyte sedimentation rate. Two blood cultures were obtained, and intravenous (IV) vancomycin was begun empirically.

**Fig. 1:**
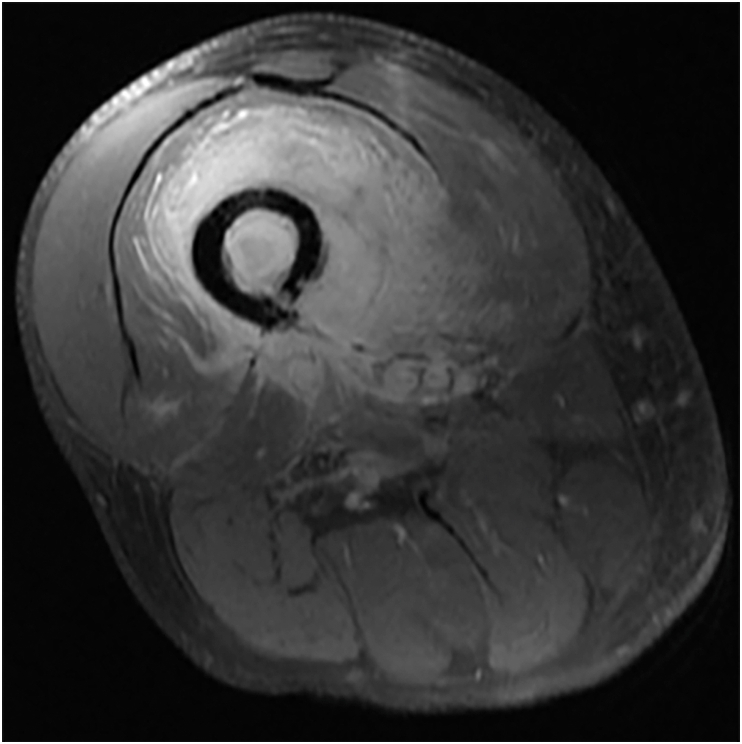
Axial T1 fat suppression post-contrast MRI at time of index presentation demonstrating right femur diaphyseal acute osteomyelitis with extraosseous abscess and pyomyositis.

A contrast enhanced magnetic resonance image (MRI) of the right thigh demonstrated a large, irregular, peripherally enhancing fluid collection centered within the vastus intermedius muscle in the mid to distal right thigh. The fluid collection wrapped around the anteromedial cortex of the femur with associated signal change within the femur (Fig. 1). IV cefepime and clindamycin were started, and vancomycin was continued. Interventional radiology successfully placed an image guided percutaneous drainage catheter into the abscess cavity. Cultured abscess fluid resulted negative, but blood cultures obtained at admission grew *S. anginosus*. The patient was discharged with a peripherally inserted central catheter for daily outpatient administration of 2 g IV ceftriaxone.

**Fig. 2:**
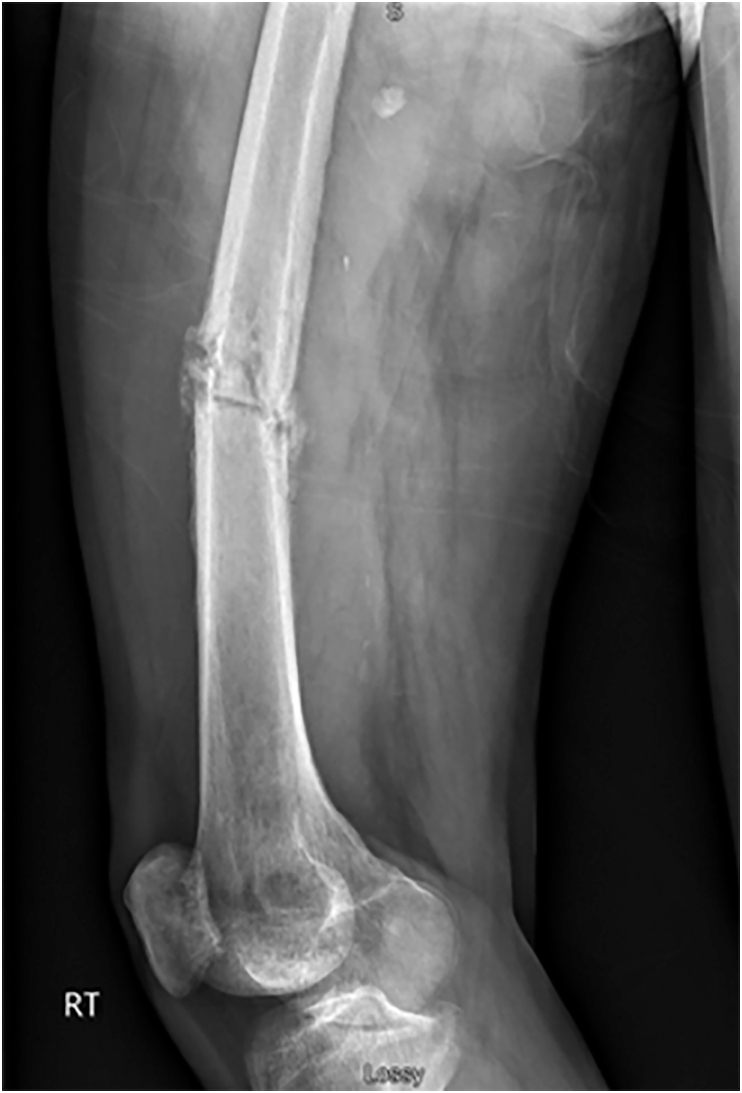
Right femur lateral radiograph demonstrating pathologic fracture through previous site of diaphyseal osteomyelitis.

Three months after discharge, the patient returned with worsening symptoms and inability to bear weight on the right leg. Radiographs revealed a pathologic fracture of the right mid-shaft femur through the site of prior diaphyseal osteomyelitis (Fig. 2). He was subsequently transferred to a tertiary university affiliated care center for further multi discipline management. An attending physician specialized in orthopaedic oncology attempted surgical stabilization with a lateral 8-hole 4.5 mm locking compression plate with bicortical 4.5 mm screws (DePuy Synthes). Open biopsies and cultures at that time did not demonstrate any recurrent organisms, and the final histology showed no evidence of neoplasm. The patient was discharged home to be followed by a local community orthopaedic surgeon.

**Fig. 3:**
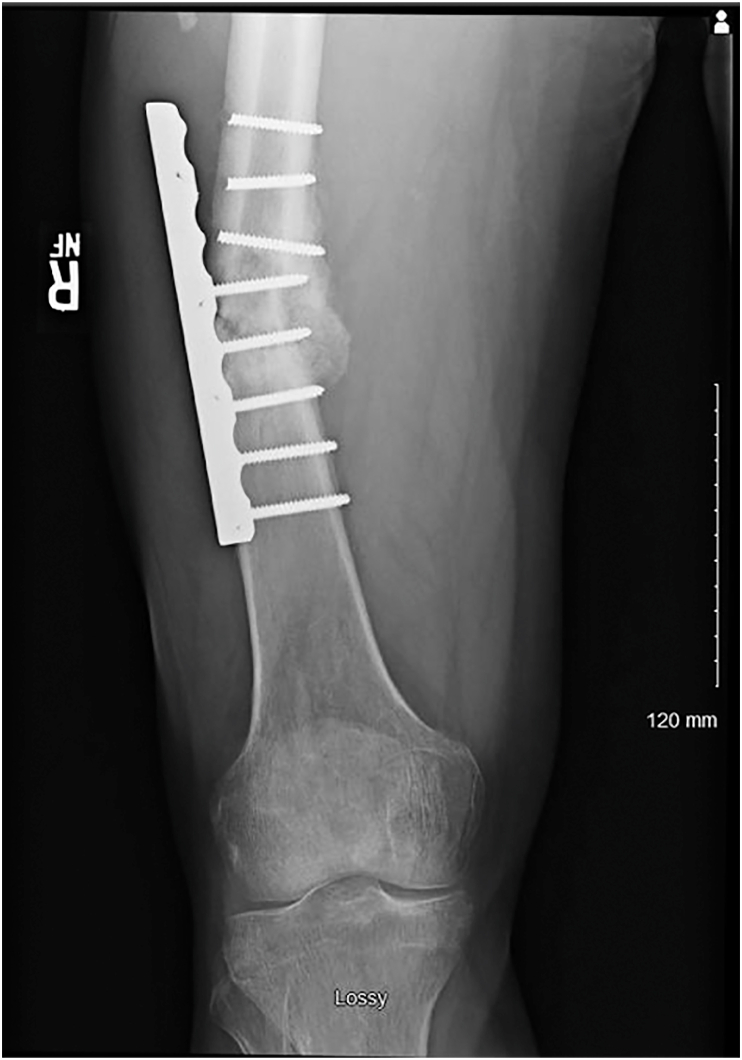
Right femur anteroposterior radiograph depicting catastrophic hardware failure of the lateral plate and screws with subsequent oligotrophic nonunion.

Five months post-operatively the patient began experiencing sharp pain in the right thigh and decreased function. Plain radiographs demonstrated catastrophic hardware failure with breakage of the proximal three screws, varus collapse of the femur, and an oligotrophic nonunion of the right femur (Fig. 3). At this time, he was referred to a local orthopaedic oncologist and traumatologist at a regional hospital. Inflammatory markers were within normal limits and his surgical wounds were completely healed without evidence of a sinus tract or drainage. Considering the patient's previous *S. anginosus* osteomyelitis, single- and two-stage procedures were discussed, and the patient elected to proceed with a single-stage procedure. The procedure was performed by the attending orthopaedic oncologist/traumatologist at the regional hospital. The patient was optimized for surgery by nil per os.

**Fig. 4:**
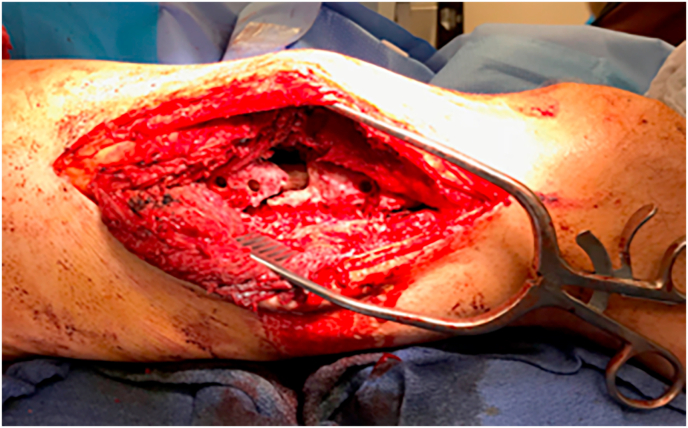
Trans vastus lateralis approach to the mid-shaft femur demonstrating fracture nonunion after the attempted stabilization with a lateral plate and screws.

**Fig. 5:**
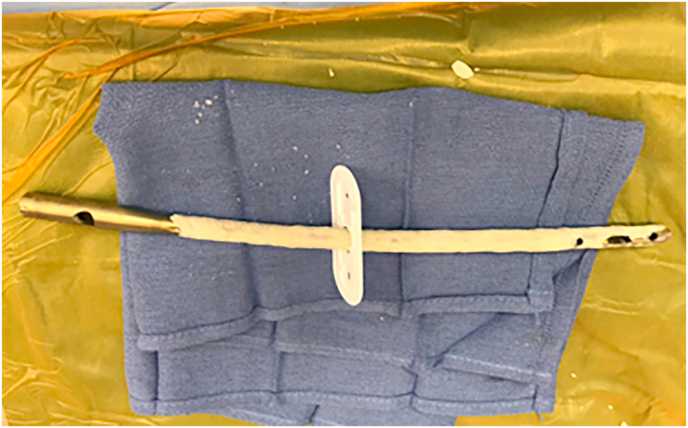
Titanium intramedullary nail coated with antibiotic-impregnated cement.

Intraoperatively, the previous lateral thigh incision was used and a trans vastus lateralis approach to the femoral diaphysis was performed (Fig. 4). The previous plate and screws were removed. Significant scar tissue and fibrinous material at the nonunion site and hypertrophic anterior and medial calluses were observed. No gross purulence or abscess was seen. The hypertrophic calluses were removed with an osteotome and rongeur. The reamer-irrigator-aspirator (RIA) device (DePuy Synthes) was used to complete endosteal preparation of the femoral canal. Given the capacious medullary canal, a 12 mm titanium intramedullary nail was selected to allow room for a coating of antibiotic-impregnated cement. Two batches of regular viscosity bone cement (Palacos) were hand mixed with 2 g of vancomycin powder and 2.4 g of tobramycin powder. The antibiotic cement delivery device was then hand placed around the exterior of the nail (Fig. 5). Iliac crest cancellous autograft was used for major bone grafting of the osseous defects at the site of nonunion. In total, the operation lasted about 90 minutes. There were no intraoperative complications or otherwise difficulties to cause deviation from the pre-operative surgical plan. The patient was discharged home one day postoperatively and advised to continue once daily 81 mg aspirin for venous thromboembolism prophylaxis.

**Fig. 6:**
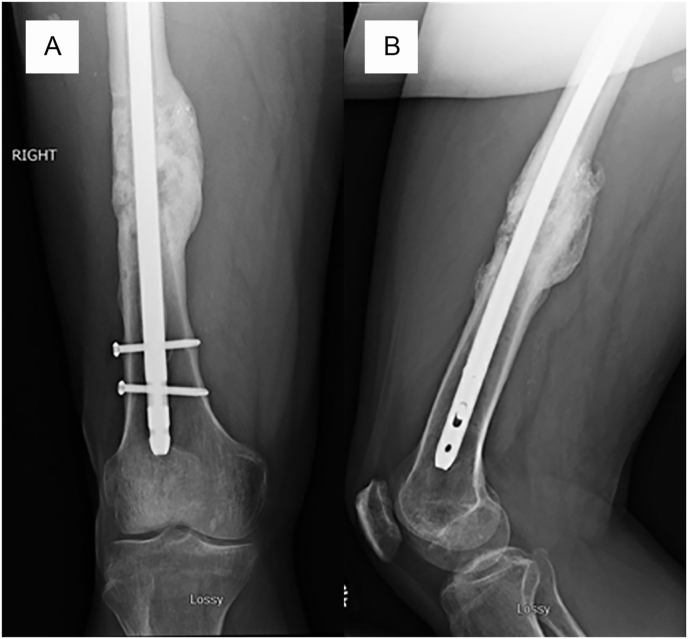
Anteroposterior (A) and lateral (B) right femur radiographs demonstrating bridging callus in 3 of 4 cortices with no evidence of catastrophic hardware failure of the antibiotic coated intramedullary nail.

Post-operatively, weightbearing was permitted as tolerated under the guidance of physical therapy. An ambulatory gait aid was used for mobility assistance. Wound healing was uneventful. Clinical and radiographic healing were achieved approximately 6 months following this revision procedure. At 12-month final follow-up, the patient was pain free, functional, and able to perform his activities of daily living without the use of a gait aid. There was no evidence of infection recurrence or implant failure (Fig. 6).

## 3 Discussion

*S. anginosus* group, formerly *Streptococcus milleri* group, is a subgroup of gram-positive catalase-negative virulent cocci belonging to the viridans streptococci group [7]. This organism is commensal to the oropharyngeal, gastrointestinal, and genitourinary flora, and thus immunosuppression or mucosal trauma (e.g., dental surgery) plays a key role in this bacterium's pathogenesis. Our patient's prior dental appointment is thought to have introduced the inciting bacterial load. One prior report described a patient with dental abscesses [2]. *S. anginosus* osteomyelitis consistently inflicts patients with comorbidities suggestive of poor bone metabolism (e.g., alcoholism or diabetes mellitus) [[2], [3], [4],[8], [9], [10], [11]].

Osteomyelitis due to *S. anginosus* can commonly present with the following signs: tenderness, swelling, erythema, insidious onset of dull pain, and with or without a fever. Late in the disease course, soft tissue abscess and discharge may occur. Due to the rarity of femoral osteomyelitis, the diagnosis can be mistaken for more common conditions. Pathologic fracture most commonly indicates a malignant process and rarely occurs due to osteomyelitis in adults aged >40 [12]. In a previous report it was noted that this infection can mimic malignancy, demonstrating symptoms such as insidious onset and profound weight loss, but a history of leukocytosis, elevated inflammatory markers, and alcohol abuse should increase a physician's index of suspicion for a *S. anginosus* musculoskeletal infection [3]. Delay in diagnosis can cause significant morbidity with potential escalation to loss of limb or life. A retrospective review demonstrated a mortality rate of 9% despite targeted susceptibility-based antibiotic therapy [13].

*S. anginosus* femoral osteomyelitis reports demonstrate a unique pattern of pathologic fracture and nonunion [[1], [2], [3], [4]]. Review of the literature also demonstrates inconsistent protocols and repeated treatment failures for cases of pathologic femur fracture secondary to *S. anginosus* osteomyelitis. The most important treatment recommendation from Vajapey et al. was bony debridement with the RIA system and an antibiotic-coated intramedullary nail [2]. It was documented that systemic antibiotic therapy for six weeks did not eliminate the patient's osteomyelitis which progressed slowly at a subclinical level, eventually resulting in a pathologic femoral shaft fracture. Vajapey et al. hypothesized that if the RIA system had been used during initial therapy, pathologic fracture may have been prevented [2]. After failed cephalomedullary nailing, Krebs et al. reported similar success with repeated use of the RIA device and antibiotic-impregnated cement rod placement [3].

Here, our patient's case displayed similar patterns to previous reports. Initial conservative management with parenteral antibiotics and percutaneous abscess drainage proved inadequate for preventing pathologic fracture. Additionally, catastrophic failure followed surgical treatment of compromised bone with a lateral based non-load bearing femoral plate and screws. Ultimate success was achieved following aggressive osseous debridement with the RIA device and antibiotic coated large diameter statically locked intramedullary nailing.

Strengths of this case include the detailed clinical course that demonstrates the morbidity associated with conservative management of *S. anginosus* femoral osteomyelitis. Moreover, definitive surgical management was achieved with good outcome, corroborating the clinical findings and therapeutic options offered by the existing literature. This case has limitations, however. Although therapeutic advice is offered, the evidence for early surgical intervention of *S. anginosus* is based in little evidence comprising case reports. Rather, this report adds a successful patient outcome to the short list of case reports with similar findings. Additionally, although the patient is now ambulating unassisted and demonstrates no residual signs of infection, long-term follow-up is needed to track potential complications in bone quality after remodeling or dormant infection.

This case involved some challenges which have also been reported in other cases involving *S. anginosus*. The rarity of this strain creates a suspicion of soft tissue sarcoma which is the common etiologic association for pathological fractures. As we reported the cultures obtained from the thigh were negative which increases the difficulty of a decisive diagnosis. Thus, the hardships in detecting of the virulent *S. anginosus* increases the likelihood of treating the infection as a mild occurrence. Therefore, physicians would tend to take the conventional route in antibiotic administration when more intensive course involving intrusive administration is more suitable. These difficulties could be overcome by taking more aggressive measures when sensing the presence of *S. anginosus* as we did in the blood cultures.

## 4 Conclusion

*S. anginosus* is a rare but serious cause of femoral osteomyelitis and can easily be missed due to its vague symptoms and overlap in presentation with other diseases. Indolent infection of this highly virulent organism can result in pathologic fracture. Significantly, this disease must be managed aggressively with surgery at onset to prevent fracture. When a large thigh abscess is documented in the setting of *S. anginosus* bacteremia, we recommend first-line management with aggressive surgical debridement involving an intraoperative site-specific antibiotic administration in the bone periphery. If pathological fracture occurs and an intramedullary nail is to be used, coating it with antibiotics has been demonstrated to result in good clinical outcome across several reports.

Upon affirming the patient's consent to publication nearly 18 months after his final operation, he wrote by e-mail (initials were anonymized and hospitals were de-identified): “I hope this information is useful to you. I want to take this opportunity to thank all of the medical personnel who helped me through this ordeal. I want to particularly thank Dr. RT and Dr. GY for their services at [the regional hospital], as well as the teams there that were involved with the JP drain and daily antibiotic infusions. Also my thanks to Dr. BN at [the university hospital] for the first surgery. Although the hardware failed, I felt the procedure was necessary to obtain accurate biopsies regarding the infection and cancer. Dr. LK was also a tremendous source of encouragement and compassion. Finally, my thanks to Dr. PW for performing the surgery that brought this ordeal to a healthy conclusion.”

## Ethical approval

The ethics review board was not required or consulted for the treatment of the patient or publication of this case.

## Sources of funding

None declared.

## Author contribution

John E. Stillson, Connor M. Bunch and Joel M. Post: Writing – Original Draft Preparation, Literature Search, Data Collection, Analysis and Interpretation.

Anthony V. Thomas, Nicolas Mjaess, Joseph A. Dynako, Andres S. Piscoya, Joel M. Post, Brian L. Ratigan, Zachary H. Goldstein: Writing – Review & Editing.

Mark M. Walsh – Supervision, Data Collection, Funding.

## Research registration

Not applicable.

## Guarantor

Mark M. Walsh is the sole guarantor of this report.

## Consent

Informed consent for publication of the manuscript and images has been provided by the patient, both verbally and written by e-mail. A copy of the written consent is available for review by the Editor-in-Chief of this journal upon request.

## Provenance and peer-review

Not commissioned, externally peer-reviewed.

## Declaration of competing interest

None declared.
